# Exact sequence variants should replace operational taxonomic units in marker-gene data analysis

**DOI:** 10.1038/ismej.2017.119

**Published:** 2017-07-21

**Authors:** Benjamin J Callahan, Paul J McMurdie, Susan P Holmes

**Affiliations:** 1 Department of Population Health and Pathobiology, NC State University, Raleigh NC, USA; 2 Whole Biome Inc, San Francisco CA, USA; 3 Department of Statistics, Stanford University, Stanford CA, USA

## Abstract

Recent advances have made it possible to analyze high-throughput marker-gene sequencing data without resorting to the customary construction of molecular operational taxonomic units (OTUs): clusters of sequencing reads that differ by less than a fixed dissimilarity threshold. New methods control errors sufficiently such that amplicon sequence variants (ASVs) can be resolved exactly, down to the level of single-nucleotide differences over the sequenced gene region. The benefits of finer resolution are immediately apparent, and arguments for ASV methods have focused on their improved resolution. Less obvious, but we believe more important, are the broad benefits that derive from the status of ASVs as consistent labels with intrinsic biological meaning identified independently from a reference database. Here we discuss how these features grant ASVs the combined advantages of closed-reference OTUs—including computational costs that scale linearly with study size, simple merging between independently processed data sets, and forward prediction—and of *de novo* OTUs—including accurate measurement of diversity and applicability to communities lacking deep coverage in reference databases. We argue that the improvements in reusability, reproducibility and comprehensiveness are sufficiently great that ASVs should replace OTUs as the standard unit of marker-gene analysis and reporting.

## Introduction

High-throughput sequencing of PCR-amplified marker genes has grown explosively over the past decade, especially as a means of taxonomically profiling microbial communities. Increasing use of marker-gene sequencing has been accompanied by increasing data set sizes; this year, we expect thousands of marker-gene studies to generate millions to billions of sequencing reads each.

The analysis of marker-gene data customarily begins with the construction of molecular operational taxonomic units (OTUs): clusters of reads that differ by less than a fixed sequence dissimilarity threshold, most commonly 3% (Westcott and Schloss, 2015; Kopylova *et al.*, 2016). The sample-by-OTU feature table serves as the basis for further analysis, with the observation of an OTU often treated as akin to the observation of a ‘species’ in the taxonomic profiling application. Many methods for defining molecular OTUs have been proposed, but the most substantive distinction is between closed-reference methods—in which reads sufficiently similar to a sequence in a reference database are recruited into a corresponding OTU—and *de novo*methods—in which reads are grouped into OTUs as a function of their pairwise sequence similarities.

Recently, new methods have been developed that resolve amplicon sequence variants (ASVs) from Illumina-scale amplicon data without imposing the arbitrary dissimilarity thresholds that define molecular OTUs (Eren *et al.*, 2013; Tikhonov *et al.*, 2015; Eren *et al.*, 2015; Callahan *et al.*, 2016a; Edgar, 2016; Amir *et al.*, 2017). ASV methods infer the biological sequences in the sample prior to the introduction of amplification and sequencing errors, and distinguish sequence variants differing by as little as one nucleotide. A similar class of methods developed for 454-scale data was typically used to ‘denoise’ sequencing data prior to constructing OTUs (Quince *et al.*, 2011), while new ASV methods are explicitly intended to replace OTUs as the atomic unit of analysis. ASV methods have demonstrated sensitivity and specificity as good or better than OTU methods and better discriminate ecological patterns (Eren *et al.*, 2013; Eren *et al.*, 2015; Callahan *et al.*, 2016a; Needham *et al.*, 2017). The higher resolution afforded by ASV methods has self-evident benefits—for example, it is clearly useful to distinguish *Neisseria gonorrhoeae* from the many other *Neisseria* species commonly found in the human microbiota—and initial evaluation has focused on that improved resolution. However, we argue here that the more important, and overlooked, advantage of ASVs is that they combine the benefits for subsequent analysis of closed-reference and de novo OTUs: ASVs are reusable across studies, reproducible in future data sets and are not limited by incomplete reference databases.

## Description of OTU and ASV methods

*De novo* OTUs are constructed by clustering sequencing reads that are sufficiently similar to one another. Many methods for constructing these clusters have been developed, but in all cases *de novo* OTUs are emergent features of a data set, with boundaries and membership that depend on the data set in which they are defined. This data set dependence is not just a practical concern: the delineation of *de novo* OTUs depends on the relative abundances of the sampled community even in the limit of infinite sequencing depth and zero errors. As a result, *de novo* OTUs defined in two different data sets cannot be compared.

Closed-reference OTUs are properties of a reference database; each reference sequence in the database defines and labels an associated closed-reference OTU. Sequencing reads are assigned to a closed-reference OTUs if they are sufficiently similar to the associated reference sequence. If the same reference database is used, closed-reference OTU assignments from independently processed data sets can be validly compared, a property we refer to as consistent labeling. However, biological variation that is not represented in the reference database is necessarily lost during assignment to closed-reference OTUs.

ASVs are inferred by a de novo process in which biological sequences are discriminated from errors on the basis of, in part, the expectation that biological sequences are more likely to be repeatedly observed than are error-containing sequences. As a result, ASV inference cannot be performed independently on each read—the smallest unit of data from which ASVs can be inferred is a sample. However, unlike *de novo* OTUs, ASVs are consistent labels because ASVs represent a biological reality that exists outside of the data being analyzed: the DNA sequence of the assayed organism. Thus, ASVs inferred independently from different studies or different samples can be validly compared.

**Figure 1:**
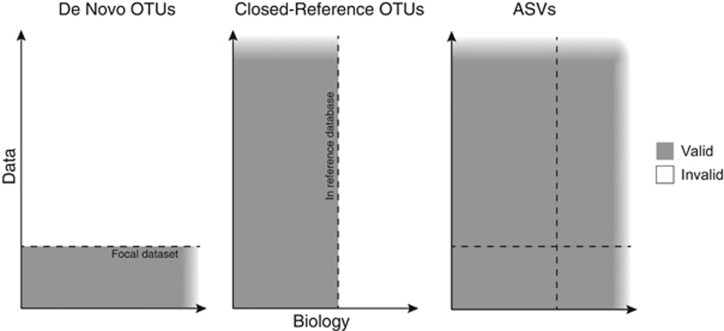
The extent of the validity of *de novo* OTUs, closed-reference OTUs and ASVs determined from a focal data set.

We schematically represent the validity of *de novo* OTUs, closed-reference OTUs and ASVs assigned from a common focal data set in Figure 1. The *x* axis represents all biological variation that exists at the sequenced genetic locus. The *y* axis represents all amplicon data generated from that locus and all future data that may be generated. The region of validity for feature type is shaded.

This schematic emphasizes the limitations inherent to both classes of OTUs. *De novo* OTUs are invalid outside of the data set in which they were defined. Closed-reference OTUs cannot capture biological variation outside of the reference database used in their construction. ASVs transcend those limitations: ASVs capture all biological variation present in the data, and ASVs inferred from a given data set can be reproduced in future data sets and validly compared between data sets.

## Practical consequences of consistent labels

### Computational tractability

Consistent labels allow the assignment of closed-reference OTUs to be split into the independent assignment of subsets of the data that are then merged together. *De novo* OTUs lack consistent labels so all data must be pooled for assignment, resulting in a number of potential sequence comparisons that scales quadratically with total sequencing effort and rendering common *de novo* methods prohibitively costly as study sizes increase (Rideout *et al.*, 2014; Mahé *et al.*, 2015).

ASV inference cannot be performed on each read independently, but can be performed on each sample independently. As long as the sequencing effort devoted to individual samples remains tractable, independent inference by sample is trivially parallelizable and enables total computation time to scale linearly and memory requirements to remain flat with increasing sample number, allowing ASVs to be inferred from arbitrarily large data sets.

### Meta-analysis

The growing number of marker-gene studies in similar environments creates opportunities for new analyses that combine studies for more power and generality. The consistent labeling of closed-reference OTUs and ASVs allows per-study tables to be merged into a cross-study table. Meta-analysis is much more difficult with *de novo* OTUs, as the raw sequence data from each study must be compiled, pooled and reprocessed into new cross-study OTUs.

### Replication

The absence of consistent labels makes replicating or falsifying previous results problematic, even impossible. Consider a significant association reported between a particular *de novo* OTU and a condition of interest. This association cannot be tested in a new data set, because that *de novo* OTU only exists within the data set in which it was defined. Therefore, results from the analysis of *de novo* OTUs can only be tested indirectly, by mapping *de novo* OTUs onto consistent labels such as taxonomy or reducing community composition to summary measures such as a diversity metric, and then testing the results at that coarser level.

### Forward prediction

A major area of translational research is the use of microbial community composition as a predictive biomarker (DiGiulio *et al.*, 2015; Baxter *et al.*, 2016). For example, the relative abundances of a set of OTUs or ASVs might be used to predict a health condition. Predictive biomarkers can be constructed through statistical methods such as regression or by various machine learning methods, and their accuracy evaluated within the study by splitting the data into training and validation subsets (Callahan *et al.*, 2016b). However, predictive biomarkers are only useful if they can be applied to new data. *De novo* OTUs exist only in the data set in which the the predictor was trained and evaluated, so predictive biomarkers based on *de novo* OTUs can’t predict from new data. Biomarkers based on consistently labeled features such as ASVs and closed-reference OTUs can be applied to new data, although closed-reference OTUs may limit predictive power by omitting predictive features absent from the reference database.

## Practical consequences of reference independence

### Diversity measurement

Reference databases are incomplete. As a result, the assignment of reads to closed-reference OTUs removes that portion of the data that is unrepresented in the reference database. This limitation is problematic if community diversity is of interest. The absence of unrepresented members of the community in closed-reference OTU tables can systematically skew diversity measures, potentially in a condition-dependent manner if some conditions are associated with a higher proportion of unrepresented taxa.

### Application across environments and genetic loci

The extent to which microbes inhabiting different environments are represented in reference databases varies greatly. In the best studied environments and for the most common genetic loci, such as 16S rRNA gene sequencing of the human gut, upwards of 90% of sequencing reads can typically be assigned to closed-reference OTUs. However, far fewer sequencing reads might be assigned to closed-reference OTUs in less-studied environments and when sequencing other genetic loci, because of limited representation in reference databases. ASVs and *de novo* OTUs more accurately represent the extent of biological variation in environments and loci without comprehensive coverage in reference databases.

### Guaranteed observation

The observation of a closed-reference OTU indicates that at least one sequencing read was sufficiently similar to the associated reference sequence to be mapped to its OTU. However, it does not guarantee that the reference sequence itself was observed—to the contrary, that is often not the case. Nevertheless, the often-absent reference sequence serves as the representative of the closed-reference OTU, which can give misleading results when downstream analyses use the unobserved representative sequences to index into other sources of data.

### Changing references

Closed-reference OTUs are independent of the data, but are wholly dependent on the set of reference sequences used in their assignment (closed-reference OTUs assigned by current methods even depend on the order of the reference sequences (Westcott and Schloss, 2015)). Therefore, closed-reference OTUs assigned against different reference databases are not comparable. In order to maintain comparability over time, reference databases must remain static or closed-reference OTU tables must be regenerated whenever a new or upgraded reference database is adopted. Because ASVs are consistent labels derived from the data alone, ASVs remain consistent into the indefinite future.

## Discussion

Molecular OTUs serve two different and orthogonal purposes. The first purpose is to translate taxonomic concepts developed in other systems into the context of high-throughput marker-gene sequencing of microbial communities by the ostensible equation of OTUs defined at certain thresholds with particular taxonomic levels (for example, 3% ribosomal OTUs are ‘like species’). The second purpose is to reduce the impact of amplicon sequencing error on measures of diversity and community composition by grouping errors together with the error-free sequence (Eren *et al.*, 2016). A consequence of this often unacknowledged dual mandate is that OTUs struggle to serve both purposes well; the connection between OTUs and species is largely unfounded (Stackebrandt and Ebers, 2006), and the most common methods often output numbers of OTUs an order of magnitude higher than the number of strains present in mock communities (Kopylova *et al.*, 2016).

Some of the shortcomings of molecular OTUs can be ameliorated. Open-reference OTU methods combine closed-reference OTU assignment with subsequent construction of *de novo* OTUs from the unassigned sequencing reads, gleaning the benefits of closed-reference OTUs without entirely sacrificing the unassignable portion of the data (Rideout *et al.*, 2014). A clever algorithm has been developed that linearizes the computational time of a special case of *de novo* OTU assignment (single-linkage clustering with a linkage threshold of 1) allowing computational tractability on extremely large data sets (Mahé *et al.*, 2015). Aggressive filtering and complete overlap between paired-end reads can reduce the rate at which OTU methods misinterpret sequencing artifacts as biological variation (Bokulich *et al.*, 2013; Kozich *et al.*, 2013; Edgar and Flyvbjerg, 2015).

Furthermore, the inference of exact sequence variants does not solve all problems. The same genome can contain multiple ASVs if there are multiple copies of the targeted genetic locus. Appealing terminology such as ‘resolution of exact sequence variants’ does not eliminate the limitations inherent to representing a complex biological organism by a short genetic barcode. For example, while necessarily better than the customary 3% ribosomal OTUs, there is still no guarantee of ecological coherence or even monophyly among genomes with the same ribosomal sequence variant (Berry *et al.*, 2017).

The merging of ASVs from different studies is attractive, but can be limited in practice. ASV tables can only be simply merged if the underlying sequence data was derived from the same genetic locus. In practice, this means that only studies using the same primer set can be simply merged, although ASVs from different-but-overlapping primer sets can be merged if the ASVs are first trimmed to the overlapping gene region. ASVs generated by different methods *can* be validly merged, but downstream analyses should account for the possibility of ‘batch effects’ due to methodological differences.

These caveats stated, the breadth of issues that ASVs cleanly solve, and the more powerful and reproducible analyses that ASVs enable, makes a dispositive case in our opinion for replacing OTUs with ASVs. ASVs have an intrinsic biological meaning as a DNA sequence. ASV inference from large marker-gene data sets is both tractable and comprehensive. ASVs improve the return-on-investment of marker-gene sequencing by better leveraging the corpus of such data sets for further discovery, especially in oft-investigated communities like those inhabiting the human body. And the ASV methods that are now available provide better resolution and accuracy than OTU methods (Eren *et al.*, 2015; Callahan *et al.*, 2016a).

For analysis to be reproducible the fundamental units must be reproducible, and *de novo* OTUs are not. For analysis to be comprehensive the fundamental units must be comprehensive, and closed-reference OTUs are not. Replacing OTUs with ASVs makes marker-gene sequencing more precise, reusable, reproducible and comprehensive. We believe that ASVs should be the standard way that marker-gene data is processed and reported going forward.

### Data Supplement

As a demonstration of reusability and computational tractability on large data sets, the DADA2 method for ASV inference was used to process the 18S rRNA gene amplicon data from the TARA Oceans project, which contains ~766 million reads (De Vargas *et al.*, 2015), on a 2016 Macbook Pro. The resulting ASV tables have been deposited at Zenodo (DOI: 10.5281/zenodo.581694) alongside the workflow scripts that generated them.
